# The Effects of Dairy Product Supplementation on Bone Health Indices in Children Aged 3 to 18 Years: A Meta-Analysis of Randomized Controlled Trials

**DOI:** 10.1016/j.advnut.2023.06.010

**Published:** 2023-07-04

**Authors:** Khemayanto Hidayat, Li-Li Zhang, René Rizzoli, Ya-Xin Guo, Yan Zhou, Yu-Jie Shi, Hong-Wen Su, Biao Liu, Li-Qiang Qin

**Affiliations:** 1 Department of Nutrition and Food Hygiene, School of Public Health, Soochow University, Suzhou, China; 2 Division of Bone Diseases, Department of Internal Medicine, Geneva University Hospitals and Faculty of Medicine, Geneva, Switzerland; 3 Branch Company, Inner Mongolia Yili Industrial Group Co, Ltd, Hohhot, China

**Keywords:** bone, dairy, milk, pediatric, growth, calcium

## Abstract

Childhood and adolescence are critical periods for optimizing skeletal growth. Dairy products are valuable sources of bone-beneficial nutrients, particularly calcium and protein. A random-effects meta-analysis of published randomized controlled trials was performed to quantitatively assess the effects of dairy supplementation on bone health indices in children and adolescents. The PubMed and Web of Science databases were searched. Dairy supplementation increased whole-body bone mineral content (BMC) (+25.37 g) and areal bone mineral density (aBMD) (+0.016 g/cm^2^), total hip BMC (+0.49 g) and aBMD (+0.013 g/cm^2^), femoral neck BMC (+0.06 g) and aBMD (+0.030 g/cm^2^), lumbar spine BMC (+0.85 g) and aBMD (+0.019 g/cm^2^), and height (0.21 cm). When expressed as a percentage difference, whole-body BMC was increased by 3.0%, total hip BMC by 3.3%, femoral neck BMC by 4.0%, lumbar spine BMC by 4.1%, whole-body aBMD by 1.8%, total hip aBMD by 1.2%, femoral neck aBMD by 1.5%, and lumbar spine aBMD by 2.6%. Dairy supplementation increased serum insulin-like growth factor I concentrations (19.89 nmol/L) and reduced concentrations of urinary deoxypyridinoline (−1.78 nmol/mmol creatinine) and serum parathyroid hormone (−10.46 pg/mL) but did not significantly affect the serum concentrations of osteocalcin, bone alkaline phosphatase, and C-terminal telopeptide of type 1 collagen. Serum 25-hydroxyvitamin D concentrations (+4.98 ng/mL) increased with vitamin D-fortified dairy supplementation. The positive effects on bone mineral mass parameters and height were generally consistent across subgroups defined by sex, geographical region, baseline calcium intake, calcium from the supplementation, trial duration, and Tanner stages. In summary, dairy supplementation during growth leads to a small but significant increase in bone mineral mass parameters, and these findings are generally supported by the changes in several biochemical parameters related to bone health.

**Statement of significance**

Dairy products are a valuable source of calcium and other bone-beneficial nutrients and, thus, are considered important for growing bones. However, the extent of the skeletal benefits of dairy products on growing bones remains debated. The present meta-analysis suggests that consuming dairy products during growth may favorably affect bone mineral mass parameters, possibly by preventing parathyroid hormone-mediated bone loss and stimulating insulin-like growth factor I secretion. Using dairy products to enrich the diet with high-quality calcium could be a fruitful dietary strategy to improve bone health during growth.

## Introduction

Bone growth begins in utero and continues toward the end of the second decade of life when the maturation process is complete and peak bone mass is achieved. Peak bone mass is generally defined as the amount of bone tissue gained when a stable skeletal state has been attained at the end of the period of growth [1]. After peak bone mass attainment, bone mass decreases with age, increasing risk of osteoporosis and subsequent fractures [1,2]. An estimated 10% increase in peak bone mass might delay the onset of osteoporosis by approximately 13 y [3]. Although between 60% and 80% of peak bone mass variance is genetically determined, environmental factors, including diet, may modify the genetic potential for skeletal growth [4,5].

Calcium, vitamin D, and protein have been identified as major dietary determinants of peak bone mass [1]. Although dairy products do not naturally contain significant amounts of vitamin D, they can be fortified with this vitamin. Dairy products are the leading natural sources of calcium and protein, accounting for ∼50% to 60% of daily calcium intake and ∼20% to 30% of daily protein intake [6]. Dairy products may influence bone mass accrual through a remodeling process mediated by calcium (ie, higher bone mass from the prevention of PTH-induced bone loss) and a modeling process through protein-stimulated IGF-I secretion, favoring periosteal expansion (ie, larger bone size) [[4], [5], [6]]. In addition, protein-stimulated IGF-I secretion may accelerate longitudinal bone growth by endochondral ossification, resulting in longer bones (ie, taller height) [[4], [5], [6]].

Over the past few decades, the effects of dairy supplementation on bone mass parameters (BMC and areal bone mineral density [aBMD]), bone turnover markers, hormones related to bone metabolism (PTH, 25(OH)D, and IGF-I), and longitudinal bone growth (height) in children and adolescents have been examined in multiple randomized controlled trials (RCTs) [[7], [8], [9], [10], [11], [12], [13], [14], [15], [16], [17], [18], [19], [20], [21], [22], [23], [24], [25], [26], [27]] with small sample sizes and inconsistent findings. Based on the understanding acquired from the available qualitative (systematic) and narrative reviews [6,[28], [29], [30], [31], [32], [33], [34]] appraising some of those RCTs, dairy products appear to have the potential to improve bone health, mainly by increasing BMC and aBMD. However, it is difficult to estimate the extent of the benefits of dairy products, as the skeletal effects of dairy supplementation were not quantitatively assessed in those reviews. To extend upon the knowledge of the role of dairy products on growing bones, a meta-analysis of published RCTs was performed to provide a quantitative estimation of the effects of dairy supplementation on bone mass parameters, bone turnover markers, hormones related to bone metabolism, and height in children and adolescents.

## Methods

The preparation and reporting of the present meta-analysis adhered to the PRISMA checklist [35]. The research question was determined by the Participants, Interventions, Comparisons, Outcomes, and Study framework. Two investigators (KH and L-LZ) independently performed the literature search, study selection, data extraction, and assessments of risk of bias (RoB) and certainty of the evidence. Disagreements between the 2 investigators were resolved by consensus.

### Study selection

The Participants, Interventions, Comparisons, Outcomes, and Study framework is shown in Table 1. Briefly, parallel or crossover RCTs that enrolled children or adolescents were included in the present meta-analysis if they met all of the following inclusion criteria: *1*) one or more intervention groups received dairy products and were compared with nondairy control (or placebo) or no intervention; *2*) reported effects on aBMD, BMC, bone formation markers (osteocalcin, bone alkaline phosphatase [BALP], procollagen type 1 N-propeptide [P1NP], and procollagen type 1 C-terminal propeptide), bone resorption markers (pyridinoline, deoxypyridinoline [Dpd], N-terminal telopeptide of type I collagen [NTx], C-terminal telopeptide of type 1 collagen [CTx], or tartrate-resistant acid phosphatase), hormones related to bone metabolism (PTH, 25(OH)D, and IGF-I), or height. For 25(OH)D, only the RCTs that used vitamin D-fortified dairy products were selected because dairy products do not naturally contain vitamin D. If multiple articles reporting findings from the same trial participants were identified, only the one with the largest sample sizes and longest trial duration or the most relevant data was included.

**TABLE 1:** Participants, Interventions, Comparisons, Outcomes, and Study design

Parameter	Criteria
Participants	Children and adolescents (≤18 y)
Intervention	Dairy products (eg, milk, yogurt, cheese)
Comparison	Nondairy placebo or control or no intervention
Outcome	Bone mineral content, areal bone mineral density, bone formation markers (osteocalcin, bone alkaline phosphatase, procollagen type 1 N-propeptide, and procollagen type 1 C-terminal propeptide), bone resorption markers (pyridinoline, deoxypyridinoline, N-terminal telopeptide of type I collagen, C-terminal telopeptide of type 1 collagen, and tartrate-resistant acid phosphatase), hormonal indices related to bone metabolism (parathyroid hormone, 25-hydroxyvitamin D, and insulin-like growth factor I), and height
Study design	Parallel or crossover randomized controlled trials

### Search strategy

The PubMed and Web of Science databases were searched for relevant RCTs reported in any language from their inception to December 2021 with no restrictions and filters, using the following combination of search terms: (milk OR cheese OR yogurt OR dairy) AND (randomized OR randomly OR trial) AND (bone OR bone remodeling OR bone resorption OR bone formation OR bone turnover OR bone mineral density OR bone mineral content OR bone mass OR bone loss OR osteoporosis OR vitamin D OR Pyridinoline OR Pyr OR deoxypyridinoline OR D-Pyr OR N-terminal telopeptide of type I collagen OR NTx OR C-terminal telopeptide of type 1 collagen OR CTx OR tartrate-resistant acid phosphatase OR TRAP OR osteocalcin OR alkaline phosphatase OR procollagen type 1 N-propeptide OR P1NP OR procollagen type 1 C-terminal propeptide OR P1CP OR parathyroid hormone OR vitamin D OR insulin-like growth factor 1 OR IGF-1 OR height). To complete the database searches, the reference lists of the retrieved articles were hand-searched for additional RCTs.

### Data extraction

Trial and participant characteristics and relevant data were extracted from each included RCT.

### Assessments of the RoB and the certainty of the evidence

The RoB of each RCT was appraised using the Cochrane Collaboration’s tool for assessing the RoB [36] that covers 6 domains of bias (each domain includes 1 or more items): selection bias (random sequence generation, allocation concealment), performance bias (blinding of the participants and personnel), detection bias (blinding of outcome assessment), attrition bias (incomplete outcome data), reporting bias (selective outcome reporting), and other bias (see below). After careful assessment, each item can be classified as “low risk,” “high risk,” or “unclear risk” of bias.

Since skeletal growth velocity and bone turnover rate vary by pubertal stage [37,38], it would be challenging to determine whether the observed changes in bone health parameters were due to puberty or dairy supplementation if the RCTs enrolled participants at pubertal age. Under the domain of other bias, all RCTs (except for those that enrolled only nonpubertal children) were assessed for the potential biasing effect of puberty. Puberty may begin between 8 and 13 y in girls and 9 and 14 y in boys [39]. Therefore, the RCTs that included children aged 8 (for trials that included girls only or both sexes) or 9 (for trials that included boys only) y and older were scrutinized for the potentially biasing effect of puberty. These RCTs required one of the following conditions to be considered to have a low RoB: *1*) enrolling only participants at the same Tanner stage, *2*) matching for Tanner stages, *3*) performing stratification by Tanner stages, or *4*) adjusting for Tanner stages statistically.

The certainty of the evidence for each outcome was evaluated using the NutriGrade [40] scoring system that includes the following items: *1*) RoB, study quality, and study limitations (maximum 3 points); *2*) precision (maximum 1 point); *3*) heterogeneity (maximum 1 point); *4*) directness (maximum 1 point); *5*) publication bias (maximum 1 point); *6*) funding bias (maximum 1 point); *7*) study design (maximum 2 points). The following total score cutoff points were assigned to define the certainty of evidence: 0 to <4 (very low), 4 to <6 (low), 6 to <8 (moderate), and ≥8 points (high).

### Statistical analyses

The 25(OH)D analysis included only vitamin D dairy products (see study selection), whereas other analyses included fortified or unfortified dairy products. The highest dose was selected if multiple doses of dairy products were assigned. If different fortification statuses of calcium or vitamin D were assigned, the one with the highest dose of additional agents (eg, dairy + 900 g of calcium was selected instead of dairy + 600 g of calcium) or those with maximal addition of agents (eg, dairy + calcium + vitamin D was selected instead of dairy + calcium) were selected. The weighted mean difference (WMD) was used as the summary measure of effect sizes (or intervention effects). A random-effects model estimated the pooled effect sizes and 95% CIs [41]. Sample size, mean difference, and SD were required from each RCT to estimate the pooled effect sizes. For parallel RCTs, the mean difference was calculated by subtracting the mean changes in bone health indices from the baseline to the end of the intervention in the control group from those in the dairy group. For crossover RCTs, the mean difference was calculated by subtracting the mean values of bone health indices at the end of the control period from those reported at the end of the dairy supplementation. If not reported, SD was calculated from the reported SE, CI, or *P* value using the standard formula [36]. To standardize results from different DXA manufacturers, the aBMD values at the hip, femoral neck, trochanter, and lumbar spine obtained by Lunar DXA or Norland DXA were converted to Hologic DXA equivalent values using published conversion equations [[42], [43], [44]]. Unfortunately, such conversion equations have not been developed for BMC (regardless of the site) and whole-body aBMD. For BMC and whole-body aBMD, we included all RCTs regardless of the DXA manufacturer. If adequate RCTs were available for each analysis (at least 10 RCTs for each analysis) [45], subgroup and meta-regression analyses were performed based on predefined factors (sex, geographical region, mean amounts of calcium and protein intakes at baseline, mean amounts of calcium and protein provided by dairy supplementation, trial duration, and Tanner stages) to identify the potential source of heterogeneity (if any) and its the influence of on the overall pooled results. In this case, subgroup and meta-regression analyses by the predefined criteria were only performed for the analyses of whole-body BMC (*n* = 10), whole-body aBMD (*n* = 10), and height (*n* = 15) because other analyses were based on a limited number (*n* < 10) of RCTs. Because there was a lack of information on the amounts of baseline protein intake and protein obtained from dairy supplementation among the included RCTs, further stratification by these factors could not be performed. The degree of heterogeneity across the included RCTs was evaluated using *I*^2^ statistics. The *I*^2^ values <25%, 25% to 50%, and >50% indicated low, moderate, and high heterogeneity, respectively [46]. Potential publication bias was evaluated using Begg’s rank correlation test and Egger’s linear regression [47]. If publication bias was evident, the trim and fill method was performed to correct the bias [48]. All statistical analyses were performed using STATA software, version 11.0 (StataCorp). All *P* values were 2-sided, and the significance level was set at < 0.05.

## Results

### Literature search

The study selection process with the reasons for exclusion is presented in Supplemental Figure 1. A total of 5379 publications were identified during the initial database searches. After duplicate removal and title/abstract review, 38 publications were retained for full-text review. The full-text review excluded 17 publications for various reasons (**Supplemental Appendix**). Finally, 21 [[7], [8], [9], [10], [11], [12], [13], [14], [15], [16], [17], [18], [19], [20], [21], [22], [23], [24], [25], [26], [27]] publications were included in the present meta-analysis.

### Trial characteristics

The characteristics of the included RCTs are reported in Supplemental Table 1. BMC and aBMD were measured by Lunar [8,10,12,15], Hologic [9,14,23,25], or Norland [13,18,27] DXA. Nine [[8], [9], [10],^13^,^15^,^17^,^18^,^24^,^25^] RCTs enrolled only girls, 1 [12] enrolled only boys, and 11 [7,11,14,16,[19], [20], [21], [22], [23],^26^,^27^] enrolled boys and girls (but sex-specific analysis was not performed). Only one trial enrolled children at preschool ages (3–5 y) [16], whereas the remaining enrolled children at elementary to high school ages (≥7–18 y) [[7], [8], [9], [10], [11], [12], [13], [14], [15],[17], [18], [19], [20], [21], [22], [23], [24], [25], [26], [27]]. Two RCTs enrolled only children at nonpubertal age ranges [11,16], 1 [19] enrolled only prepubertal children (Tanner stage 1), 3 [8,12,24] enrolled only pubertal children (Tanner stage ≥2), 8 [9,10,13,15,17,18,23,27] enrolled prepubertal and pubertal children (Tanner stage ≥1), and 7 [7,14,[20], [21], [22],^25^,^26^] enrolled some or all children at pubertal age ranges but did not report the information on puberty status or stage. The changes in bone health indices were adjusted for Tanner stages at baseline in several RCTs [[13], [14], [15],^17^,^18^,^27^], whereas few other RCTs specifically mentioned that the participants were at similar Tanner stage [8,19] or stratified by [9] or matched [10] by Tanner stages at baseline.

The trial duration was ≥1 y in 12 [[7], [8], [9], [10], [11],[13], [14], [15],^17^,^18^,^23^,^24^] RCTs and <1 y in 9 [12,16,[19], [20], [21], [22],[25], [26], [27]] RCTs. In all RCTs, participants in the dairy product group were asked to consume dairy products in addition to their habitual diet. Fifteen [[7], [9],[11], [12], [13], [14],[17], [18], [19], [20], [21], [22],[25], [26], [27]] RCTs supplemented the participants with milk, 4 [[8], [10], [23], [24]] with various dairy products, 1 [15] with cheese, and 1 [16] with yogurt. Most RCTs asked the control group participants to continue their habitual diet, whereas a few RCTs assigned unfortified juice [12] or a placebo [15,22] as controls. Information on habitual dairy intake was rarely reported. All RCTs did not disclose the amount of protein obtained from the supplementation. Not all RCTs provided data on the amount of calcium provided by dairy supplementation intake or on baseline intakes of calcium, protein, and vitamin D. Among the RCTs that reported the data, dairy products intake provided 150 to 1723 mg of calcium a day [[8], [9], [10],[12], [13], [14], [15], [16], [17], [18], [19], [20],[22], [23], [24], [25],^27^]; baseline calcium intake was higher in Western participants [[8], [9], [10],^12^,^15^,[19], [23]] (ranging from 664–1470 mg/d) than in Asian participants [13,14,[16], [17], [18],^25^,^27^] (ranging from 150–494 mg/d); baseline intake of protein was relatively adequate (ranging from 45.8–100.6 g/d) [[8], [9], [10],[12], [13], [14], [15], [16], [17], [18],^20^,[23], [24]], whereas baseline vitamin D intake was mostly low (<10 μg/d) [8,10,[12], [13], [14], [15],^17^,^18^,^25^].

### RoB

The RoB assessment is reported in Supplemental Table 2. Only a few RCTs had adequately disclosed information on random sequence generation (3 of 21 RCTs) and allocation concealment (1 of 21 RCTs). Although the trial’s participants, investigators, and outcome assessors were rarely blinded among the included RCTs, risk of performance bias and detection bias in all RCTs was deemed as low because the outcomes were based on objective measurements (ie, bone mass parameters, bone turnover markers, hormones, and height), which were unlikely to be influenced by the lack of blinding. For incomplete outcome data, an attrition rate of 20% was used as a cutoff point. The attrition rates in milk and control groups after the randomization were <20% (low risk) in 15 RCTs, >20% (high risk) in 1 RCT, and not reported (unclear risk) in 5 RCTs. Risk of reporting bias in all RCTs was judged as unclear, as selective outcome reporting could not be ruled out due to the unavailability of trial protocols.

With respect to other bias, the potential biasing effect of puberty was assessed. Nineteen [[7], [8], [9], [10],[12], [13], [14], [15],[17], [18], [19], [20], [21], [22], [23], [24], [25], [26], [27]] RCTs included some or all children within or above puberty age ranges, and the information on Tanner stages was reported in 12 [[8], [9], [10],^12^,^13^,^15^,[17], [18], [19],^23^,^24^,^27^] RCTs and not reported in 7 [7,14,[20], [21], [22],^25^,^26^] RCTs. Among the RCTs that reported the information on Tanner stages, risk of other bias was low in 9 RCTs that took Tanner stages into account in their study design (by restriction [8,19], matching [10], or stratification [9]) or analysis (by statistical adjustment [13,15,17,18,27]) and high in 3 [12,23,24] RCTs that did not account for Tanner stages in their study design or analysis. Among the RCTs that did not report the information on Tanner stages, 1 [14] RCT was considered to have a low RoB because Tanner stages were taken into account in its analysis (by statistical adjustment), and 6 [7,[20], [21], [22],^25^,^26^] RCTs were considered to have an unclear RoB because they did not consider Tanner stages in their study design or analysis. Two [11,16] RCTs that did not include children at pubertal ages were judged to have a low RoB.

### Meta-analyses

#### Bone mineral mass parameters

##### Main analysis

**FIGURE 1:**
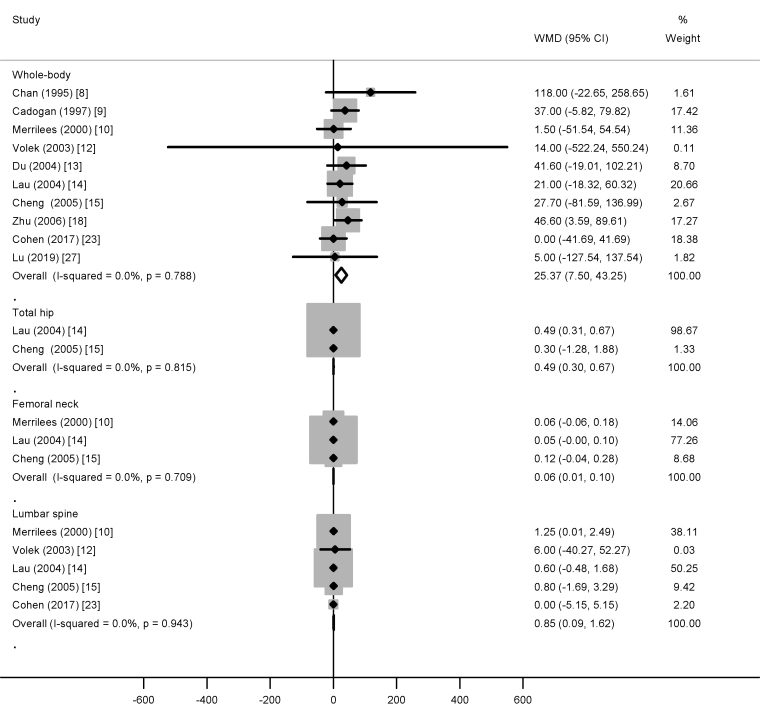
The weighted mean difference (WMD) (95% CI) in bone mineral content between dairy product and control groups in children/adolescents. All data are expressed in g.

**FIGURE 2:**
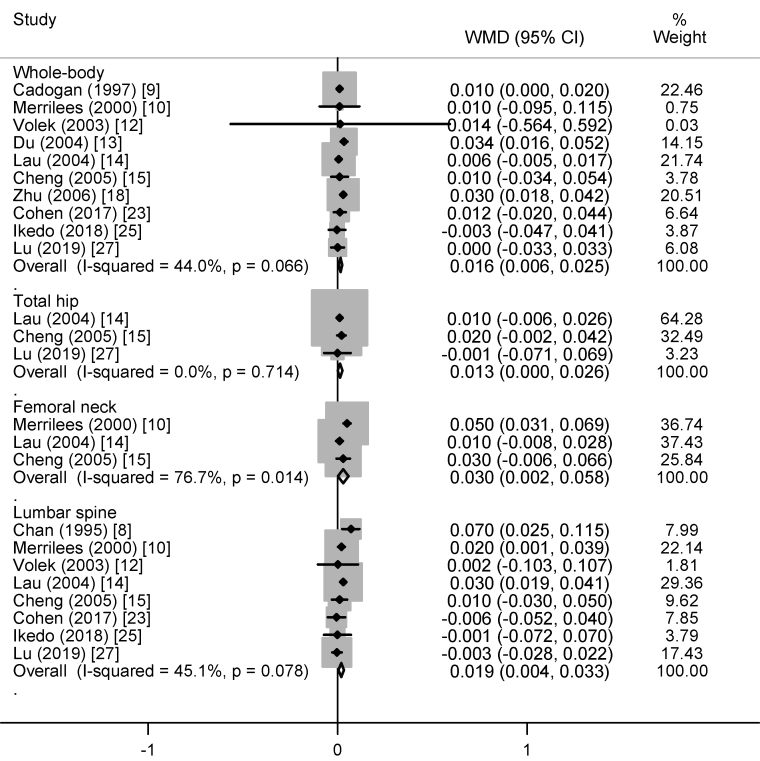
The weighted mean difference (WMD) (95% CI) in bone mineral density between dairy product and control groups in children/adolescents. All data are expressed in g/cm^2^.

Compared with controls, dairy supplementation increased whole-body BMC (25.37 g; 95% CI: 7.50, 43.25 g), total hip BMC (0.49 g; 95% CI: 0.30, 0.67 g), femoral neck BMC (0.06 g; 95% CI: 0.01, 0.10 g), and lumbar spine BMC (0.85 g; 95% CI: 0.09, 1.62 g) (Figure 1). Similarly, whole-body aBMD (0.016 g/cm^2^; 95% CI: 0.006, 0.025 g/cm^2^), total hip aBMD (0.013 g/cm^2^; 95% CI: 0.000, 0.026 g/cm^2^), femoral neck aBMD (0.030 g/cm^2^; 95% CI: 0.002, 0.058), and lumbar spine aBMD (0.019 g/cm^2^; 95% CI: 0.004, 0.033 g/cm^2^) were also increased with dairy supplementation (Figure 2). No heterogeneity (*I*^2^ = 0%) was observed in the analyses of BMC (regardless of the site) and hip BMD, whereas moderate-to-high heterogeneity (^2^ ≥ 44%) was observed in the analyses of other outcomes. There was no evidence of publication bias for all outcomes (all *P* values for Egger’s ≥0.32; all *P* values for Begg’s ≥0.37).

When expressed as a percentage difference, whole-body BMC was increased by 3.0%, total hip BMC by 3.3%, femoral neck BMC by 4.0%, lumbar spine BMC by 4.1%, whole-body aBMD by 1.8%, total hip aBMD by 1.2%, femoral neck aBMD by 1.5%, and lumbar spine aBMD by 2.6% (Supplemental Figure 2).

##### Subgroup and meta-regression analyses

A significant increase in whole-body BMC and aBMD was observed in the participants with lower baseline calcium intake (<700 mg/d), with a lower amount of calcium from dairy supplementation (<1000 mg/d), in the RCTs that were performed in Asian countries (mainly China), in the RCTs that enrolled only girls, in the RCTs with longer trial duration (≥1 y), and when Tanner stages were considered in the study design or analysis (Supplemental Table 3). However, meta-regression analyses did not indicate sex, geographical region, the mean amounts of baseline calcium intake and calcium provided by dairy supplementation, trial duration, and Tanner stages as the sources of heterogeneity and effect modifiers (all *P* values for meta-regression ≥0.16; Supplemental Table 3).

#### Height

##### Main analysis

**FIGURE 3:**
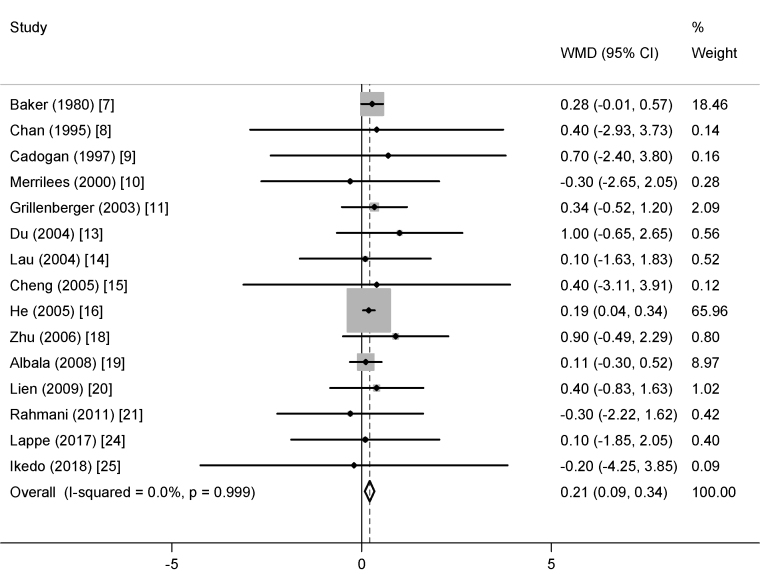
The weighted mean difference (WMD) (95% CI) in height between dairy product and control groups in children/adolescents. All data are expressed in cm.

Children in the dairy group had a larger increase in height (0.21 cm; 95% CI: 0.09, 0.34 cm) than those in the control group (Figure 3), with no heterogeneity (*I*^2^ = 0%). There was no indication of publication bias (all *P* Egger’s ≥0.88; all *P* Begg’s ≥0.59).

##### Subgroup and meta-regression analyses

The increase in height was only significant in the participants with higher mean calcium intake at baseline (≥700 mg/d), with a lower mean amount of calcium from dairy supplementation (<1000 mg/d), in the RCTs that were performed in Asian countries (mainly China), in the RCTs that enrolled both sexes, in the RCTs with longer trial duration (≥1 y), and when Tanner stages were not considered in the study design or analysis (Supplemental Table 3). However, meta-regression analyses revealed that sex, geographical region, the mean amounts of baseline calcium intake and calcium provided by dairy supplementation, trial duration, and Tanner stages were not significant effect modifiers of the effect of dairy supplementation on height (Supplemental Table 3).

#### Biochemistry

**FIGURE 4:**
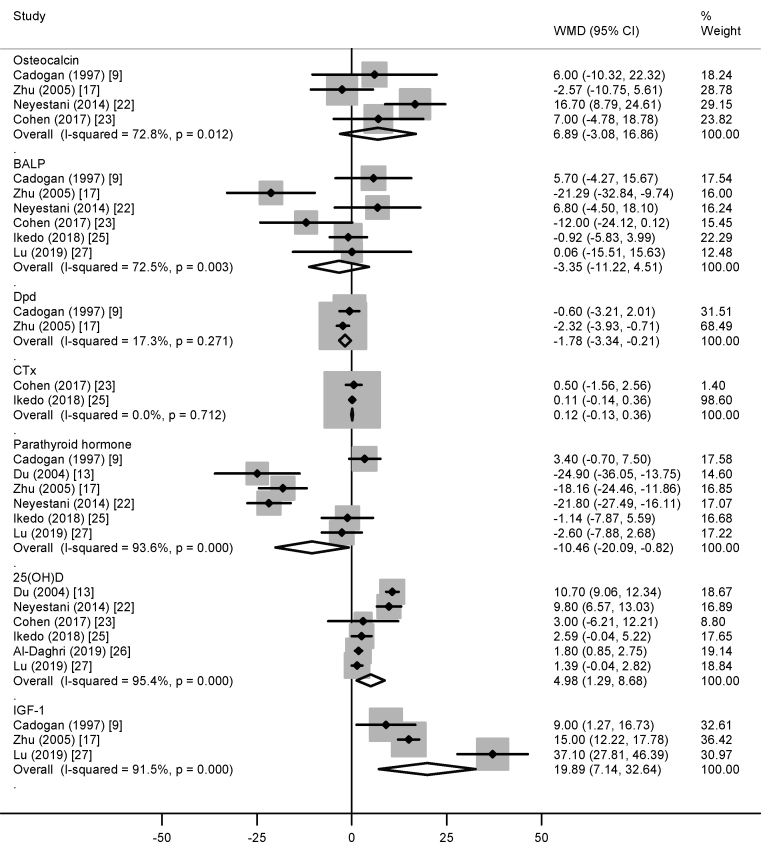
The weighted mean difference (WMD) (95% CI) in the concentrations of osteocalcin, bone alkaline phosphatase (BALP), deoxypyridinoline (Dpd), C-terminal telopeptide of type 1 collagen (CTx), parathyroid hormone, 25-hydroxyvitamin D (25(OH)D), and IGF-I between dairy product and control group in children/adolescents. The data are expressed in ng/mL for osteocalcin, CTx, and 25(OH)D; μg/L for BALP; nmol/mmol creatinine for Dpd; pg/mL for parathyroid hormone; and nmol/L for IGF-I.

The difference in the concentrations of serum osteocalcin (6.89 ng/mL; 95% CI: −3.08, 16.86 ng/mL), BALP (−3.35 μg/L; 95% CI: −11.22, 4.51 μg/L), and CTx (0.12 ng/mL; 95% CI: −0.13, 0.36 ng/mL) between the dairy and control groups was not significant (Figure 4). The dairy group had a greater increase in serum IGF-I concentrations (19.89 nmol/L; 95% CI: 7.14, 32.64 nmol/L) and greater reductions in concentrations of urinary Dpd (−1.78 nmol/mmol creatinine; 95% CI: −3.34, −0.21 nmol/mmol creatinine) and serum PTH (−10.46 pg/mL; 95% CI: −20.09, −0.82 pg/mL) than the control group (Figure 4). Serum 25(OH)D concentrations (4.98 ng/mL; 95% CI: 1.29, 8.68 ng/mL; Figure 4) were higher in the vitamin D-fortified dairy group than in the control group. Low heterogeneity was observed in the analyses of Dpd and Ctx (*I*^2^ ≤ 17.3%), whereas high heterogeneity was evident in the analyses of other outcomes (*I*^2^ ≥ 72.5%).

### Certainty of the evidence

The certainty of the evidence was high for height and whole-body BMC and aBMD, moderate for total hip aBMD, lumbar spine BMC and aBMD, BALP, Dpd, PTH, 25(OH)D, and IGF-I, and low for other outcomes (Supplemental Table 4).

## Discussion

### Summary of primary findings

The present meta-analysis of published RCTs suggests that dairy supplementation increased all the investigated bone mineral mass parameters, height, and IGF-I concentrations and reduced the concentrations of Dpd and PTH in children/adolescents. The reduction in bone resorption marker Dpd without affecting bone formation markers and the reduction in PTH concentrations are excellent evidence of the beneficial effect of increased calcium intake in preventing PTH-induced bone loss (resorption) due to inadequate calcium intake [49]. The increase in concentrations of IGF-I, a growth hormone important in muscle mass maintenance, skeletal growth, and bone mass acquisition, is consistent with the anabolic effect of protein ingestion [[49], [50], [51]]. Improved vitamin D status (reflected by increased 25[OH]D concentrations) aligns with the increased vitamin D intake from vitamin D-fortified dairy products and allows optimal calcium absorption [49,52].

To the best of our knowledge, this is the first meta-analysis to report the effects of dairy supplementation on various bone health indices in children and adolescents. A previous meta-analysis [28] that pooled 9 RCTs on calcium supplementation and 4 on dairy supplementation (3 [9,13,14] of which were eligible for the present meta-analysis) together in a single analysis showed a null effect of the combined supplementation on whole-body BMC in children. However, a significant increase in whole-body BMC was observed in the RCTs [9,13,14] that enrolled participants with lower baseline calcium intake, possibly because calcium functions as a threshold nutrient, meaning that bone mass increases as calcium intake increases up to the putative threshold, above which excess calcium is excreted rather than contributing to bone mass [53]. We found that the increase in whole-body BMC and aBMD was only significant when the mean calcium intake at baseline was lower (<700 mg/d) but not higher (≥700 mg/d), which agreed with the previous meta-analysis. Interestingly, whole-body BMC and aBMD were only significantly increased in Asian RCTs, but not Western ones, and baseline calcium intake was typically higher in the latter than in the former. Furthermore, the skeletal response to dairy supplementation appeared to depend on baseline calcium intake rather than the amount of calcium provided by dairy supplementation. In this case, we found that lower (<1000 mg/d) but not higher (≥1000 mg/d) doses of calcium supplementation significantly increased whole-body BMC and aBMD. Further review of the individual RCTs included in both subgroups revealed that baseline calcium intake was relatively high in the RCTs with a higher dose of calcium and low in those with a lower dose of calcium. It should be acknowledged that although the subgroup findings by the amounts of baseline calcium intake and calcium provided by dairy supplementation and geographical region provided a crude indication of the potential threshold effect of calcium, meta-regression analyses did not indicate these factors as significant effect modifiers. Moreover, the increase in height was only significant when the mean calcium intake at baseline was higher (≥700 mg/d) but not lower (<700 mg/d), which did not agree with the potential threshold effect of calcium. Further clarification on the potential effect modification by calcium intake is warranted.

Adequate calcium intake throughout the lifespan is recommended to maximize peak bone mass during growth, maintain peak bone mass during adulthood, and prevent age- or menopause-related bone loss during older age [1,54]. Although dairy supplementation improved bone mineral mass parameters in children/adolescents, the magnitude of the improvement was very small. Therefore, it remains unknown whether such a small improvement in bone mass parameters could prevent fracture. The findings of observational studies on the association between dairy/milk consumption and risk of fracture in children/adolescents have been inconsistent, with some [[55], [56], [57]] suggesting that milk consumption is not associated with risk of fracture and others suggesting that milk avoidance [[58], [59], [60]] or low milk consumption [61,62] is associated with a higher risk of fracture. Despite the uncertainty of whether the observed positive effects on bone mass parameters confer a lower fracture risk, some [[63], [64], [65]], but not all [66], observational studies indicated the potential long-term skeletal benefits of dairy consumption during earlier life. Although several RCTs [[67], [68], [69]] found that the beneficial effects of calcium or milk supplementation on bone mass parameters were sustained after the supplementation was discontinued, others [17,70,71] did not find such sustained effects. Notably, dietary calcium intake was maintained at postsupplemented levels after the discontinuation of supplementation in the RCTs that demonstrated sustained effects. By comparison, dietary calcium intake was returned to presupplemented levels after the discontinuation of supplementation in the RCTs that showed transient effects.

The present meta-analysis was subject to some caveats that warrant cautious interpretation. First, although the favorable effects of dairy supplementation on bone mass parameters were statistically significant, the effects appeared small in magnitude and were of subtle clinical significance. Therefore, more evidence is needed to substantiate our encouraging findings. Second, only limited RCTs were available for each outcome. Even for the outcomes with the most trials, only 10 RCTs were available. The limited number of RCTs included in each analysis hampered the robustness of the overall findings and the ability to fully evaluate the potential source of heterogeneity and effect modifiers through comprehensive subgroup and meta-regression analyses. Finally, although we did not find any evidence of publication bias, the assessment of publication bias is often underpowered when less than 10 RCTs are included in a single analysis. Therefore, publication bias could still have existed for the outcomes with less than 10 RCTs. The present findings could have been seriously affected if RCTs with null or unfavorable results were not published.

In summary, dairy supplementation during growth leads to a small but significant increase in bone mineral mass parameters, and these findings are generally supported by the changes in several biochemical parameters related to bone health. Large, long-term multicenter RCTs are warranted to investigate whether the effects of dairy supplementation on bone health indices differ according to relevant factors that could modify the supplementation effects (eg, race/ethnicity, calcium intake, protein intake, vitamin D status).

## Author contributions

The authors’ responsibilities were as follows—KH: designed the research and wrote the paper; KH, L-LZ: performed the literature search, data extraction, and data analyses; KH, RZ: interpreted the data; Y-XG: created figures and tables; KH, L-LZ, RZ, Y-XG: revised the paper; KH, RZ, Y-JS, H-WS, BL, L-QQ: took primary responsibility for the final and intellectual content; and all authors: read and approved the final manuscript.

### Conflict of interest

Y-JS, H-WS, and BL are employed by Yili Group, a dairy product producer. All other authors report no conflicts of interest.

### Funding

This research was supported by the National Natural Science Foundation of China (no. 82173502 to L-QQ and no. 82150410453 to KH).

## References

[1] Weaver C.M., Gordon C.M., Janz K.F., Kalkwarf H.J., Lappe J.M., Lewis R. (2016). The National Osteoporosis Foundation's position statement on peak bone mass development and lifestyle factors: a systematic review and implementation recommendations. Osteoporos. Int..

[2] Heaney R.P., Abrams S., Dawson-Hughes B., Looker A., Marcus R., Matkovic V. (2000). Peak bone mass. Osteoporos. Int..

[3] Hernandez C.J., Beaupré G.S., Carter D.R. (2003). A theoretical analysis of the relative influences of peak BMD, age-related bone loss and menopause on the development of osteoporosis. Osteoporos. Int..

[4] Rizzoli R., Bianchi M.L., Garabédian M., McKay H.A., Moreno L.A. (2010). Maximizing bone mineral mass gain during growth for the prevention of fractures in the adolescents and the elderly. Bone.

[5] Rizzoli R., Biver E., Brennan-Speranza T.C. (2021). Nutritional intake and bone health. Lancet Diabetes Endocrinol.

[6] Rizzoli R. (2014). Dairy products, yogurts, and bone health. Am. J. Clin. Nutr..

[7] Baker I.A., Elwood P.C., Hughes J., Jones M., Moore F., Sweetnam P.M. (1980). A randomised controlled trial of the effect of the provision of free school milk on the growth of children. J. Epidemiol. Community Health.

[8] Chan G.M., Hoffman K., McMurry M. (1995). Effects of dairy products on bone and body composition in pubertal girls. J. Pediatr..

[9] Cadogan J., Eastell R., Jones N., Barker M.E. (1997). Milk intake and bone mineral acquisition in adolescent girls: randomised, controlled intervention trial. BMJ.

[10] Merrilees M.J., Smart E.J., Gilchrist N.L., Frampton C., Turner J.G., Hooke E. (2000). Effects of diary food supplements on bone mineral density in teenage girls. Eur. J. Nutr..

[11] Grillenberger M., Neumann C.G., Murphy S.P., Bwibo N.O., van’t Veer P., Hautvast J.G. (2003). Food supplements have a positive impact on weight gain and the addition of animal source foods increases lean body mass of Kenyan schoolchildren. J. Nutr..

[12] Volek J.S., Gómez A.L., Scheett T.P., Sharman M.J., French D.N., Rubin M.R. (2003). Increasing fluid milk favorably affects bone mineral density responses to resistance training in adolescent boys. J. Am. Diet. Assoc..

[13] Du X., Zhu K., Trube A., Zhang Q., Ma G., Hu X. (2004). School-milk intervention trial enhances growth and bone mineral accretion in Chinese girls aged 10–12 years in Beijing. Br. J. Nutr..

[14] Lau E.M., Lynn H., Chan Y.H., Lau W., Woo J. (2004). Benefits of milk powder supplementation on bone accretion in Chinese children. Osteoporos. Int..

[15] Cheng S., Lyytikäinen A., Kröger H., Lamberg-Allardt C., Alén M., Koistinen A. (2005). Effects of calcium, dairy product, and vitamin D supplementation on bone mass accrual and body composition in 10–12-y-old girls: a 2-y randomized trial. Am. J. Clin. Nutr..

[16] He M., Yang Y.X., Han H., Men J.H., Bian L.H., Wang G.D. (2005). Effects of yogurt supplementation on the growth of preschool children in Beijing suburbs, Biomed. Environ. Sci..

[17] Zhu K., Du X., Cowell C.T., Greenfield H., Blades B., Dobbins T.A. (2005). Effects of school milk intervention on cortical bone accretion and indicators relevant to bone metabolism in Chinese girls aged 10–12 y in Beijing. Am. J. Clin. Nutr..

[18] Zhu K., Zhang Q., Foo L.H., Trube A., Ma G., Hu X. (2006). Growth, bone mass, and vitamin D status of Chinese adolescent girls 3 y after withdrawal of milk supplementation. Am. J. Clin. Nutr..

[19] Albala C., Ebbeling C.B., Cifuentes M., Lera L., Bustos N., Ludwig D.S. (2008). Effects of replacing the habitual consumption of sugar-sweetened beverages with milk in Chilean children. Am. J. Clin. Nutr..

[20] do Lien T.K., Nhung B.T., Khan N.C., le Hop T., Nga N.T., Hung N.T. (2009). Impact of milk consumption on performance and health of primary school children in rural Vietnam, Asia Pac. J. Clin. Nutr..

[21] Rahmani K., Djazayery A., Habibi M.I., Heidari H., Dorosti-Motlagh A.R., Pourshahriari M. (2011). Effects of daily milk supplementation on improving the physical and mental function as well as school performance among children: results from a school feeding program. J. Res. Med. Sci..

[22] Neyestani T.R., Hajifaraji M., Omidvar N., Nikooyeh B., Eshraghian M.R., Shariatzadeh N. (2014). Calcium-vitamin D-fortified milk is as effective on circulating bone biomarkers as fortified juice and supplement but has less acceptance: a randomised controlled school-based trial. J. Hum. Nutr. Diet.

[23] Cohen T.R., Hazell T.J., Vanstone C.A., Rodd C., Weiler H.A. (2017). Bone health is maintained, while fat mass is reduced in pre-pubertal children with obesity participating in a 1-year family-centered lifestyle intervention. Calcif. Tissue Int..

[24] Lappe J.M., McMahon D.J., Laughlin A., Hanson C., Desmangles J.C., Begley M. (2017). The effect of increasing dairy calcium intake of adolescent girls on changes in body fat and weight. Am. J. Clin. Nutr..

[25] Ikedo A., Arimitsu T., Kurihara T., Ebi K., Fujita S. (2018). The effect of ongoing vitamin D and low-fat milk intake on bone metabolism in female high-school endurance runners. J. Clin. Med. Res..

[26] Al-Daghri N.M., Amer O.E., Khattak M.N.K., Sabico S., Ghouse Ahmed Ansari M., Al-Saleh Y. (2019). Effects of different vitamin D supplementation strategies in reversing metabolic syndrome and its component risk factors in adolescents. J. Steroid Biochem. Mol. Biol..

[27] Lu J.X., Pan H., Hu X.Q., Huang Z.W., Zhang Q. (2019). Effects of milk powder intervention on bone mineral density and indicators related to bone metabolism in Chinese adolescents. Osteoporos. Int..

[28] Huncharek M., Muscat J., Kupelnick B. (2008). Impact of dairy products and dietary calcium on bone-mineral content in children: results of a meta-analysis. Bone.

[29] Wallace T.C., Bailey R.L., Lappe J., O’Brien K.O., Wang D.D., Sahni S. (2021). Dairy intake and bone health across the lifespan: a systematic review and expert narrative. Crit. Rev. Food Sci. Nutr..

[30] Iuliano S., Hill T.R. (2019). Dairy foods and bone health throughout the lifespan: a critical appraisal of the evidence. Br. J. Nutr..

[31] de Lamas C., de Castro M.J., Gil-Campos M., Gil Á., Couce M.L., Leis R. (2019). Effects of dairy product consumption on height and bone mineral content in children: a systematic review of controlled trials. Adv. Nutr..

[32] Kouvelioti R., Josse A.R., Klentrou P. (2017). Effects of dairy consumption on body composition and bone properties in youth: a systematic review. Curr. Dev. Nutr..

[33] van den Heuvel E.G.H.M., Steijns J.M.J.M. (2018). Dairy products and bone health: how strong is the scientific evidence?. Nutr. Res. Rev..

[34] Rizzoli R. (2022). Dairy products and bone health. Aging Clin. Exp. Res..

[35] Moher D., Liberati A., Tetzlaff J., Altman D.G., PRISMA Group (2009). Preferred reporting items for systematic reviews and meta-analyses: the PRISMA statement. PLOS Med.

[36] Higgins J., Thomas J. (2020). https://training.cochrane.org/handbook.

[37] Rizzoli R., Bonjour J.P. (1999). Determinants of peak bone mass and mechanisms of bone loss. Osteoporos. Int..

[38] Bonjour J.P., Chevalley T. (2014). Pubertal timing, bone acquisition, and risk of fracture throughout life. Endocr. Rev..

[39] Farello G., Altieri C., Cutini M., Pozzobon G., Verrotti A. (2019). Review of the literature on current changes in the timing of pubertal development and the incomplete forms of early puberty. Front. Pediatr..

[40] Schwingshackl L., Knüppel S., Schwedhelm C., Hoffmann G., Missbach B., Stelmach-Mardas M. (2016). Perspective: NutriGrade: a scoring system to assess and judge the meta-evidence of randomized controlled trials and cohort studies in nutrition research. Adv. Nutr..

[41] DerSimonian R., Laird N. (1986). Meta-analysis in clinical trials, Control. Clin. Trials.

[42] Wilson K.E. Practical considerations when replacing a DXA system. https://hologiced.com/library/practical-considerations-when-replacing-a-dxa-system/.

[43] Genant H.K., Grampp S., Glüer C.C., Faulkner K.G., Jergas M., Engelke K. (1994). Universal standardization for dual x-ray absorptiometry: patient and phantom cross-calibration results. J. Bone Miner. Res..

[44] Lu Y., Fuerst T., Hui S., Genant H.K. (2001). Standardization of bone mineral density at femoral neck, trochanter and Ward’s triangle. Osteoporos. Int..

[45] Higgins J.P., Thompson S.G., Deeks J.J., Altman D.G. (2011). https://handbook-5-1.cochrane.org/chapter_9/9_6_5_1_ensure_that_there_are_adequate_studies_to_justify.htm.

[46] Higgins J.P., Thompson S.G., Deeks J.J., Altman D.G. (2003). Measuring inconsistency in meta-analyses. BMJ.

[47] Egger M., Davey Smith G., Schneider M., Minder C. (1997). Bias in meta-analysis detected by a simple, graphical test. BMJ.

[48] Duval S., Tweedie R. (2000). Trim and fill: a simple funnel-plot-based method of testing and adjusting for publication bias in meta-analysis. Biometrics.

[49] Hidayat K., Chen J.S., Wang T.C., Liu Y.J., Shi Y.J., Su H.W. (2022). The effects of milk supplementation on bone health indices in adults: a meta-analysis of randomized controlled trials. Adv. Nutr..

[50] Giustina A., Mazziotti G., Canalis E. (2008). Growth hormone, insulin-like growth factors, and the skeleton. Endocr. Rev..

[51] Bonjour J.P. (2016). The dietary protein, IGF-I, skeletal health axis. Horm. Mol. Biol. Clin. Investig..

[52] Whiting S.J., Calvo M.S. (2021). Vitamin D: nutrition information brief. Adv. Nutr..

[53] Heaney R.P. (2009). Dairy and bone health. J. Am. Coll. Nutr..

[54] Mitchell P.J., Cooper C., Dawson-Hughes B., Gordon C.M., Rizzoli R. (2015). Life-course approach to nutrition. Osteoporos. Int..

[55] Ma D., Jones G. (2004). Soft drink and milk consumption, physical activity, bone mass, and upper limb fractures in children: a population-based case-control study. Calcif. Tissue Int..

[56] Petridou E., Karpathios T., Dessypris N., Simou E., Trichopoulos D. (1997). The role of dairy products and non alcoholic beverages in bone fractures among schoolage children. Scand. J. Soc. Med..

[57] Allison R.M., Birken C.S., Lebovic G., Howard A.W., L’Abbe M.R., Morency M.E. (2020). Consumption of cow’s milk in early childhood and fracture risk: a prospective cohort study. Am. J. Epidemiol..

[58] Black R.E., Williams S.M., Jones I.E., Goulding A. (2002). Children who avoid drinking cow milk have low dietary calcium intakes and poor bone health. Am. J. Clin. Nutr..

[59] Goulding A., Rockell J.E., Black R.E., Grant A.M., Jones I.E., Williams S.M. (2004). Children who avoid drinking cow’s milk are at increased risk for prepubertal bone fractures. J. Am. Diet. Assoc..

[60] Konstantynowicz J., Nguyen T.V., Kaczmarski M., Jamiolkowski J., Piotrowska-Jastrzebska J., Seeman E. (2007). Fractures during growth: potential role of a milk-free diet. Osteoporos. Int..

[61] Pires L.A., Souza A.C., Laitano O., Meyer F. (2005). Bone mineral density, milk intake and physical activity in boys who suffered forearm fractures. J. Pediatr. (Rio J).

[62] Manias K., McCabe D., Bishop N. (2006). Fractures and recurrent fractures in children; varying effects of environmental factors as well as bone size and mass. Bone.

[63] Murphy S., Khaw K.T., May H., Compston J.E. (1994). Milk consumption and bone mineral density in middle aged and elderly women. BMJ.

[64] Opotowsky A.R., Bilezikian J.P. (2003). Racial differences in the effect of early milk consumption on peak and postmenopausal bone mineral density. J. Bone Miner. Res..

[65] Kalkwarf H.J., Khoury J.C., Lanphear B.P. (2003). Milk intake during childhood and adolescence, adult bone density, and osteoporotic fractures in US women. Am. J. Clin. Nutr..

[66] Feskanich D., Bischoff-Ferrari H.A., Frazier A.L., Willett W.C. (2014). Milk consumption during teenage years and risk of hip fractures in older adults. JAMA Pediatr.

[67] Daly R.M., Petrass N., Bass S., Nowson C.A. (2008). The skeletal benefits of calcium- and vitamin D3-fortified milk are sustained in older men after withdrawal of supplementation: an 18-mo follow-up study. Am. J. Clin. Nutr..

[68] Ting G.P., Tan S.Y., Chan S.P., Karuthan C., Zaitun Y., Suriah A.R. (2007). A follow-up study on the effects of a milk supplement on bone mineral density of postmenopausal Chinese women in Malaysia. J. Nutr. Health Aging.

[69] Bonjour J.P., Chevalley T., Ammann P., Slosman D., Rizzoli R. (2001). Gain in bone mineral mass in prepubertal girls 3.5 years after discontinuation of calcium supplementation: a follow-up study. Lancet.

[70] Lee W.T., Leung S.S., Leung D.M., Cheng J.C. (1996). A follow-up study on the effects of calcium-supplement withdrawal and puberty on bone acquisition of children. Am. J. Clin. Nutr..

[71] Lee W.T., Leung S.S., Leung D.M., Wang S.H., Xu Y.C., Zeng W.P. (1997). Bone mineral acquisition in low calcium intake children following the withdrawal of calcium supplement. Acta Paediatr.

