# Supplementary material for: The Effects of Dairy Product Supplementation on Bone Health Indices in Children Aged 3 to 18 Years: A Meta-Analysis of Randomized Controlled Trials
Source: Adv Nutr. 2023 Jul 4;14(5):1187–96. doi: 10.1016/j.advnut.2023.06.010 (PMC10509403; doi:10.1016/j.advnut.2023.06.010)
Supplement: Multimedia component 1 [file mmc1.docx]

**The effects of dairy product supplementation on bone health indices in children aged 3 to 18 years: A meta-analysis of randomized controlled trials**

Khemayanto Hidayat et al.

5739 publications identified during initial database searches:

- PubMed database (n = 1737)

- Web of Science database (n = 4002)

Duplicate removed (n = 2786)

2953 publications

Excluded by abstracts/titles (n = 2915)

Articles selected for full-text evaluation (n = 38)

17 publications were excluded:

- Dairy (intervention) vs dairy (control) (n = 10)

- Non-randomized trials (n = 5)

- BMC was presented in g/year (n = 1)

- Postprandial trial (n = 1)

21 publications were included in the present meta-analysis

**Supplementary Figure 1** Flow-chart of the study selection process

**Supplementary Appendix** The list of the excluded studies

Dairy (intervention) vs. dairy (control)

1. Courteix D, Jaffré C, Lespessailles E, Benhamou L. Cumulative effects of calcium supplementation and physical activity on bone accretion in premenarchal children: a double-blind randomised placebo-controlled trial. Int J Sports Med 2005;26(5):332-8.
2. Ma XM, Huang ZW, Yang XG, Su YX. Calcium supplementation and bone mineral accretion in Chinese adolescents aged 12-14 years: a 12-month, dose-response, randomised intervention trial. Br J Nutr 2014;112(9):1510-1520.
3. Zhang ZQ, Ma XM, Huang ZW, Yang XG, Chen YM, Su YX. Effects of milk salt supplementation on bone mineral gain in pubertal Chinese adolescents: a 2-year randomized, double-blind, controlled, dose-response trial. Bone 2014;65:69-76.
4. Gibbons MJ, Gilchrist NL, Frampton C, Maguire P, Reilly PH, March RL, Wall CR. The effects of a high calcium dairy food on bone health in pre-pubertal children in New Zealand. Asia Pac J Clin Nutr 2004;13(4):341-347.
5. St-Onge MP, Goree LL, Gower B. High-milk supplementation with healthy diet counseling does not affect weight loss but ameliorates insulin action compared with low-milk supplementation in overweight children. J Nutr 2009;139(5):933-938.
6. Zhang ZQ, Ma XM, Huang ZW, Yang XG, Chen YM, Su YX. Effects of milk salt supplementation on bone mineral gain in pubertal Chinese adolescents: a 2-year randomized, double-blind, controlled, dose-response trial. Bone 2014;65:69-76.
7. Brett NR, Lavery P, Agellon S, Vanstone CA, Maguire JL, Rauch F, Weiler HA. Dietary vitamin D dose-response in healthy children 2 to 8 y of age: a 12-wk randomized controlled trial using fortified foods. Am J Clin Nutr.2016;103(1):144-152.
8. Ganmaa D, Stuart JJ, Sumberzul N, Ninjin B, Giovannucci E, Kleinman K, Holick MF, Willett WC, Frazier LA, Rich-Edwards JW. Vitamin D supplementation and growth in urban Mongol school children: Results from two randomized clinical trials. PLoS One 2017;12(5):e0175237.
9. Petrova D, Bernabeu Litrán MA, García-Mármol E, Rodríguez-Rodríguez M, Cueto-Martín B, López-Huertas E, Catena A, Fonollá J. Еffects of fortified milk on cognitive abilities in school-aged children: results from a randomized-controlled trial. Eur J Nutr 2019;58(5):1863-1872.
10. Marwaha RK, Dabas A, Puri S, Kalaivani M, Dabas V, Yadav S, Dang A, Pullakhandam R, Gupta S, Narang A. Efficacy of Daily Supplementation of Milk Fortified With Vitamin D2 for Three Months in Healthy School Children: A Randomized Placebo Controlled Trial. Indian Pediatr 2021;58(9):820-825.

Non-randomized trials

1. Lampl M, Johnston FE. The effects of protein supplementation on the growth and skeletal maturation of New Guinean school children. Ann Hum Biol 1978;5(3):219-227.
2. Renner E , Hermes M, Stracke H. Bone mineral density of adolescents as affected by calcium intake through milk and milk products. Int Dairy J 1998;8:759-764.
3. Hoppe C, Mølgaard C, Juul A, Michaelsen KF. High intakes of skimmed milk, but not meat, increase serum IGF-I and IGFBP-3 in eight-year-old boys. Eur J Clin Nutr 2004;58(9):1211-1216.
4. Kristensen M, Jensen M, Kudsk J, Henriksen M, Mølgaard C. Short-term effects on bone turnover of replacing milk with cola beverages: a 10-day interventional study in young men. Osteoporos Int 2005;16(12):1803-1808.
5. Budek AZ, Hoppe C, Michaelsen KF, Mølgaard C. High intake of milk, but not meat, decreases bone turnover in pre-pubertal boys after 7 days. Eur J Clin Nutr 2007;61(8):957-962.

BMC was presented in g/year

1. Vogel KA, Martin BR, McCabe LD, Peacock M, Warden SJ, McCabe GP, Weaver CM. The effect of dairy intake on bone mass and body composition in early pubertal girls and boys: a randomized controlled trial. Am J Clin Nutr 2017;105(5):1214-1229.

Postprandial trial

1. Weaver CM, Campbell WW, Teegarden D, Craig BA, Martin BR, Singh R, Braun MM, Apolzan JW, Hannon TS, Schoeller DA, DiMeglio LA, Hickey Y, Peacock M. Calcium, dairy products, and energy balance in overweight adolescents: a controlled trial. Am J Clin Nutr 2011;94(5):1163-1170.

**Supplementary Table 1** Characteristics of the included randomized controlled trials

| Reference | Age, years | Female, % | Duration, months | Intervention (no. participants) | Dairy dose | Vitamin D fortification | Calcium from dairy, mg/d | Baseline calcium intake,  mg/d | Baseline protein intake,  g/d | Baseline vitamin D intake, μg/d | Baseline serum 25(OH)D, ng/ml | Outcome | Tanner stages at baseline and the end of the trial | Whether Tanner stages were considered in the study design or analysis | DXA manufactures |
| --- | --- | --- | --- | --- | --- | --- | --- | --- | --- | --- | --- | --- | --- | --- | --- |
| Baker et al., 1980, UK (7) | 7-9 | 48 | 21.5 | Fluid milk (281)  vs  No milk (239) | 190 ml/d | Not fortified | NR | NR | NR | NR | NR | Height | NR | Not considered in the study design or analysis | ─ |
| Chan et al. 1995, USA (8) | 11 (mean) | 100 | 12 | Dairy (24)  vs  No dairy (24) | Not specified | Likely to be fortified | ≥1200 | 1470 | 70 | 7.2 | NR | BMC (whole- body), aBMD (lumbar spine) and height | Baseline: stage 2 | All participants were at the same Tanner stage at baseline | Lunar |
| Cadogan et al., 1997, UK (9) | 12 (mean) | 100 | 18 | Fluid milk (44)  vs  No Milk (38) | 568 ml/d | Not fortified | 567 | 738 | 59.1 | NR | NR | aBMD (whole-body), BMC (whole-body), OC, BALP, NTx, D-Pyr, PTH, and IGF-1 | Baseline: stages 1-4 | Stratification by Tanner stages was performed | Hologic |
| Merrilees et al., 2000, New Zealand (10) | 15-16 | 100 | 24 | Dairy (46)  vs  No dairy (45) | Not specified | Not fortified | ≥1000 | 744.1 | 62.5 | 1.91 | NR | aBMD (whole-body, femoral neck, trochanter, and lumbar spine), BMC (whole-body, femoral neck, trochanter, and lumbar spine), and height | Baseline: stages 1 and 2 | All participants were matched for the Tanner stage at baseline | Lunar |
| Grillenberger et al., 2003, Kenya (11) | 6-7 (median: 7.1) | 47 | 23 | Fluid milk (132)  vs  No Milk (117) | Not specified | Not fortified | NR | NR | NR | NR | NR | Height | Not within the puberty age range | Not within the puberty age range | ─ |
| Volek et al. 2003, USA (12) | 13-17 (mean: 14) | 0 | 3 | Fluid milk (14)  vs  Unfortified apple or  grape juice (14) | ≥ 708 ml/d | Likely to be fortified | 1723 | ≥ 912 | 100.6 | 10.4 | NR | aBMD (whole-body and lumbar spine) and BMC (whole-body and lumbar spine) | Baseline: stages 3 and 4 | Not considered in the study design or analysis | Lunar |
| Du et al., 2004, China (13) | 10-12 (mean: 10) | 100 | 24 | Ca and vit D- fortified  fluid milk  (113; included) or  Ca-fortified fluid  milk (111)  vs  No milk (122) | 330 ml/d | Fortified | 560 | 418 | 53.1 | 0.89 | 20.6 | aBMD (whole-body  ), BMC (whole-body), PTH, and  25(OH)D | Baseline: Stages 1-4 | Adjusted | Norland |
| Lau et al., 2004, China (14) | 9-10 (mean: 10) | 45 | 18 | 80 g milk (102;  included) powder  or 40 g milk  powder (100)  vs  No milk (122) | 80 g/d | Fortified | 1300 | 494 | 86 | 0.78 | NR | aBMD (whole-body, femoral neck, hip, and lumbar spine), BMC (whole-body, femoral neck, hip, and lumbar spine), and height | NR | Adjusted | Hologic |
| Cheng et al., 2005, Finland (15) | Mean: 11 | 100 | 24 | Cheese (39)  vs  Placebo pills (39) | 210 g/d | Not fortified | 1000 | 664 | 14.9 (% of energy) | 2.7 | NR | aBMD (whole-body, femoral neck, hip, and lumbar spine) and BMC (whole-body, femoral neck, hip, and lumbar spine) | Baseline: Stages 1 and 2 | Adjusted | Lunar |
| He et al., 2005, China (16) | 3-5 (mean: 4) | 46 | 9 | Yogurt (201)  vs  No yogurt (201) | 125 g/d | Not fortified | 150 | 348.3 | 45.8 | NR | NR | Height | Not within the puberty age range | Not within the puberty age range | ─ |
| Zhu et al. 2005, China (17) | 10-12 (mean: 10) | 100 | 24 | Ca and vit D-fortified  fluid milk  (210; included) or  Ca-fortified fluid  milk (177)  vs  No milk (219) | 330 ml/d | Fortified | 560 | 420 | 54 | 1.03 | 41.2 | OC, BALP, D-Pyr, PTH,  and IGF-1 | Baseline: Stages 1-3  At the end of intervention: Stages: 1-5 | Adjusted | ─ |
| Zhu et al. 2006, China (18) | 10-12 (mean: 10) | 100 | 24 | Ca and vit D-fortified  fluid milk  (210; included) or  Ca-fortified fluid  milk (177)  vs  No milk (219) | 330 ml/d | Fortified | 560 | 420 | 54 | 1.03 | 41.2 | aBMD (whole-body), BMC (whole-body), and height | Baseline: Stages 1-3  At the end of intervention: Stages: 1-5 | Adjusted | Norland |
| Albala et al., 2008, Chile (19) | 8-10 (mean: 9) | 41 | 4 | Fluid milk (47)  vs  No milk (46) | 600 ml/d | Not fortified | 960 | 983.7 | NR | NR | NR | Height | At baseline: stage 1 | All participants were at the same Tanner stage at baseline | ─ |
| Lien et al., 2009, Vietnam (20) | 7-8 | 52.2 | 6 | Ca and vit D-fortified  fluid milk  (150; included) or  fluid  milk (151)  vs  No milk (143) | 500 ml/d | Fortified | 780 | NR | 51.4 | NR | NR | Height | NR | Not considered in the study design or analysis | ─ |
| Rahmani et al., 2011, Iran (21) | Mean: 9 | 51 | 3 | Fluid milk (47)  vs  No milk (46) | 250 ml/d | Not fortified | NR | NR | NR | NR | NR | Height | NR | Not considered in the study design or analysis | ─ |
| Neyestani et al., 2013, Iran (22) | 9-12 | 54 | 3 | Ca and vit D-fortified  fluid milk  (80; included) or  fluid milk (80)  vs  Placebo (53) | 200 ml/d | Fortified | 500 | NR | NR | NR | 23.8 | OC, BALP,  and 25(OH)D | NR | Not considered in the study design or analysis | ─ |
| Cohen et al., 2017, Canada (23) | 6-8 (mean: 7.8) | 56 | 12 | Dairy (47)  vs  No dairy (46) | 2 servings/d | Likely to be fortified | < 1000 | ≥ 700 | 68.4 | NR | NR | aBMD (whole-body and lumbar spine), BMC (whole-body and lumbar spine), OC, BALP, CTx, and PTH | At baseline: Stages 1 and 2 | Pre-pubertal | Hologic |
| Lappe et al., 2017, USA (24) | 13-14 | 100 | 12 | Dairy (136)  vs  No dairy (138) | NR | Likely to be fortified | 1200 | 552 | 14.8 (% of energy) | NR | NR | Height | At baseline: stage 5 | Not considered in the study design or analysis | ─ |
| Ikedo et al., 2018, Japan (25) | Mean: 16 | 100 | 6 | Fluid milk +  vit D pills (10)  vs  No milk (10) | 200 ml/d | Not fortified (but vitamin D was obtained from the pills) | 315 | 445 | NR | 10.5 | NR | aBMD (whole-body and lumbar spine), BALP, CTx,  PTH,  25(OH)D, and height | NR | Not considered in the study design or analysis | Hologic |
| Al-Daghri et al., 2019, Saudi Arabia (26) | 12-18 (mean: 15) | 51 | 6 | Vit D fortified-fluid milk  (18)  vs  No milk (22) | 200 ml/d | Fortified | NR | NR | NR | NR | < 20 | 25(OH)D | NR | Not considered in the study design or analysis | ─ |
| Lu et al., 2019, China (27) | 12-15 (mean: 13) | 50 | 6 | Vit D-fortified milk  powder + 300 g Ca  (43; included), 600  g Ca (54), or 900 g  Ca (48)  vs  No milk (62) | 40 g/d | Fortified | 900 | 363.5 | NR | NR | 11.8 | BMC (whole-body), BMD (whole-body, hip, and lumbar spine), BALP, TRAP,  PTH, 25(OH)D,  and IGF-1 | At baseline: 1-5 | Adjusted | Norland |

25(OH)D, 25-hydroxyvitamin D; aBMD, areal bone mineral density; BALP, bone alkaline phosphatase; BMC, bone mineral content; CTx, C-terminal telopeptide of type 1 collagen; D-Pyr deoxypyridinoline; IGF-1, insulin-like growth factor 1; NR not reported; NTx, N-terminal telopeptide of type I collagen; OC, osteocalcin; PTH, parathyroid hormone; P1NP procollagen type 1 N-propeptide; TRAP tartrate-resistant acid phosphatase

**Supplementary Table 2** The methodological quality of the included randomized controlled trials

| **Study(year)** | **Selection bias** | | **Performance bias** | **Detection bias** | **Attrition bias** | **Reporting bias** | **Other bias** |
| --- | --- | --- | --- | --- | --- | --- | --- |
|  | **Random sequence generation** | **Allocation**  **concealment** | **Blinding of participants and**  **personnel** | **Blinding of outcome assessment** | **Incomplete outcome data** | **Selective reporting** |  |
| Baker et al., 1980, UK (7) | Unclear | Unclear | Low risk | Low risk | Unclear | Unclear | Unclear |
| Chan et al. 1995, USA (8) | Unclear | Unclear | Low risk | Low risk | Low risk | Unclear | Low |
| Cadogan et al., 1997, UK (9) | Low risk | Unclear | Low risk | Low risk | Low risk (2.4) | Unclear | Low |
| Merrilees et al., 2000, New Zealand (10) | Unclear | Unclear | Low risk | Low risk | Low risk (14) | Unclear | Low |
| Grillenberger et al., 2003, Kenya (11) | Unclear | Unclear | Low risk | Low risk | Low risk (11) | Unclear | Low |
| Volek et al. 2003, USA (12) | Unclear | Unclear | Low risk | Low risk | Unclear (0) | Unclear | High |
| Du et al., 2004, China (13) | Unclear | Unclear | Low risk | Low risk | Low risk (7.8) | Unclear | Low |
| Lau et al., 2004, China (14) | Unclear | Unclear | Low risk | Low risk | Low risk (5.8) | Unclear | Low |
| Cheng et al., 2005, Finland (15) | Low risk | Low risk | Low risk | Low risk | Low risk (11) | Unclear | Low |
| He et al., 2005, China (16) | Unclear | Unclear | Low risk | Low risk | Unclear | Unclear | Low |
| Zhu et al. 2005, China (17) | Unclear | Unclear | Low risk | Low risk | Low risk (7.8) | Unclear | Low |
| Zhu et al. 2006, China (18) | Unclear | Unclear | Low risk | Low risk | Low risk (7.8) | Unclear | Low |
| Albala et al., 2008, Chile (19) | Low risk | Unclear | Low risk | Low risk | Low risk (5) | Unclear | Low |
| Lien et al., 2009, Vietnam (20) | Unclear | Unclear | Low risk | Low risk | Unclear | Unclear | Unclear |
| Rahmani et al., 2011, Iran (21) | Unclear | Unclear | Low risk | Low risk | Unclear | Unclear | Unclear |
| Neyestani et al., 2013, Iran (22) | Unclear | Unclear | Low risk | Low risk | Low risk (8) | Unclear | Unclear |
| Cohen et al., 2017, Canada (23) | Unclear | Unclear | Low risk | Low risk | Low risk (7) | Unclear | High |
| Lappe et al., 2017, USA (24) | Unclear | Unclear | Low risk | Low risk | Low risk (2) | Unclear | High |
| Ikedo et al., 2018, USA (25) | Unclear | Unclear | Low risk | Low risk | High risk (31) | Unclear | Unclear |
| Al-Daghri et al., 2019, Saudi Arabia (26) | Unclear | Unclear | Low risk | Low risk | Low risk (0) | Unclear | Unclear |
| Lu et al., 2019, China (27) | Unclear | Unclear | Low risk | Low risk | Low risk (10.7) | Unclear | Low |

**Supplementary Figure 2** The weighted mean difference (95% CI [confidence interval]) in areal bone mineral density (aBMD) and bone mineral content (BMC) between dairy product and control groups in children/adolescents. All data are expressed in %.

**Supplementary Table 3** Subgroup and meta-regression analyses of the effects of dairy supplementation on whole-body bone mineral content (BMC) and areal bone mineral density (aBMD) and height in children and adolescents aged 3 to 18 years

|  | Whole-body BMC | | | |  | Whole-body aBMD | | | |  | Height | | | |
| --- | --- | --- | --- | --- | --- | --- | --- | --- | --- | --- | --- | --- | --- | --- |
|  | n | Effect size (95% CI), values in g | *I*^2^ | *P* value for meta-regression |  | n | Effect size (95% CI), values in g/cm^2^ | *I*^2^ | *P* value for meta-regression |  | n | Effect size (95% CI), values in cm | *I*^2^ | *P* value for meta-regression |
| Overall | 10 | 25.37 (7.50, 43.25)* | 0 | ─ |  | 10 | 0.016 (0.006, 0.025)* | 44 | ─ |  | 15 | 0.21 (0.09, 0.34)* | 0 | ─ |
| Mean baseline Ca intake |  |  |  |  |  |  |  |  |  |  |  |  |  |  |
| ≥ 700 mg/d | 5 | 17.47 (-8.10, 43.03) | 0 | 0.42 |  | 4 | 0.010 (-0.001, 0.021) | 0 | 0.58 |  | 7 | 0.20 (0.05, 0.35)* | 0 | 0.50 |
| < 700 mg/d | 5 | 32.93 (7.93, 57.93)* | 0 |  |  | 6 | 0.017 (0.003, 0.031)* | 65.1 |  |  | 4 | 0.11 (-0.29, 0.51) | 0 |  |
| Not reported | 0 | Not available | ─ |  |  | 0 | Not available | ─ |  |  | 4 | 0.28 (0.02, 0.54)* | 0 |  |
| Mean Ca from dairy |  |  |  |  |  |  |  |  |  |  |  |  |  |  |
| ≥ 1000 mg/d | 5 | 19.69 (-9.93, 49.31) | 0 | 0.65 |  | 4 | 0.006 (-0.004, 0.016) | 0 | 0.31 |  | 5 | 0.08 (-0.95, 1.10) | 0 | 0.47 |
| < 1000 mg/d | 5 | 28.63 (6.21, 51.04)* | 0 |  |  | 6 | 0.019 (0.007, 0.031)* | 54.3 |  |  | 7 | 0.20 (0.06, 0.34)* | 0 |  |
| Not reported | 0 | ─ | ─ |  |  | 0 | ─ | ─ |  |  | 3 | 0.27 (0.01, 0.55)* | 0 |  |
| Geographical region |  |  |  |  |  |  |  |  |  |  |  |  |  |  |
| Western countries | 6 | 18.00 (-6.89, 42.89) | 0 | 0.43 |  | 5 | 0.010 (-0.001, 0.020) | 0 | 0.53 |  | 7 | 0.22 (-0.01, 0.45) | 0 | 0.83 |
| Asian countries | 4 | 33.22 (7.54, 58.90)* | 0 |  |  | 5 | 0.017 (0.002, 0.033)* | 71.8 |  |  | 7 | 0.20 (0.05, 0.35)* | 0 |  |
| Others | 0 | Not available | ─ |  |  | 0 | Not available | ─ |  |  | 1 | Not pooled | ─ |  |
| Sex |  |  |  |  |  |  |  |  |  |  |  |  |  |  |
| Girls | 6 | 35.45 (12.19, 58.71)* | 0 | 0.22 |  | 6 | 0.027 (0.018, 0.036)* | 0 | 0.16 |  | 8 | 0.56 (-0.21, 1.34) | 0 | 0.39 |
| Boys | 1 | Not pooled | ─ |  |  | 1 | Not pooled | ─ |  |  | 0 | Not available | ─ |  |
| Both sexes | 3 | 10.84 (-17.12, 38.80) | 0 |  |  | 3 | 0.006 (-0.004, 0.016) | 0 |  |  | 7 | 0.20 (0.08, 0.33)* | 0 |  |
| Trial duration |  |  |  |  |  |  |  |  |  |  |  |  |  |  |
| ≥ 1 y | 8 | 25.76 (7.71, 43.81)* | 0 | 0.77 |  | 7 | 0.018 (0.007, 0.028)* | 57.7 | 0.28 |  | 10 | 0.31 (0.06, 0.57)* | 0 | 0.54 |
| < 1 y | 2 | 5.52 (-123.15, 134.18) | 0 |  |  | 3 | 0.001 (-0.028,0.025) | 0 |  |  | 5 | 0.18 (-0.04, 0.32) | 0 |  |
| Tanner stages^1^ |  |  |  |  |  |  |  |  |  |  |  |  |  |  |
| Considered in the study design or analysis | 8 | 31.11 (11.31, 50.91)* | 0 | 0.22 |  | 7 | 0.017 (0.006, 0.028)* | 60.1 | 0.54 |  | 7 | 0.57 (-0.20, 1.34) | 0 | 0.65 |
| Not considered | 2 | 0.08 (-41.48, 41.65) | 0 |  |  | 3 | 0.007 (-0.019, 0.033) | 0 |  |  | 8 | 0.20 (0.08, 0.33)* | 0 |  |

An asterisk (*) indicates a statistically significant effect (*P* < 0.05). ^1^Applies only to the trials that enrolled participants within the age range of 8 (for trials that included girls only or both sexes) or 9 (for trials that included boys only) years to ≥ 13 years

**Supplementary Table 4** The certainty of the evidence for the effect of milk supplementation on each outcome in children and adolescents aged 3 to 18 years

|  | Risk of bias, study quality, and study limitations | Precision | Heterogeneity | Directness | Publication bias | Funding bias | Study design | Total score | Quality of evidence |
| --- | --- | --- | --- | --- | --- | --- | --- | --- | --- |
| Whole-body BMC | 2.25 | 1 | 1 | 1 | 1 | 0 | 2 | 8.25 | High |
| Total hip BMC | 2.25 | 0 | 0 | 1 | 0 | 0 | 2 | 5.25 | Low |
| Femoral neck BMC | 2.25 | 0 | 0 | 1 | 0 | 0 | 2 | 5.25 | Low |
| Lumbar spine BMC | 2.25 | 1 | 0.5 | 1 | 0.5 | 0 | 2 | 7.25 | Moderate |
| Whole-body aBMD | 2.25 | 1 | 1 | 1 | 1 | 0 | 2 | 8.25 | High |
| Total hip aBMD | 2.25 | 1 | 0 | 1 | 0 | 0 | 2 | 6.25 | Moderate |
| Femoral neck aBMD | 2.25 | 0 | 0 | 1 | 0 | 0 | 2 | 5.25 | Low |
| Lumbar spine aBMD | 2.25 | 1 | 0.3 | 1 | 0.5 | 0 | 2 | 7.05 | Moderate |
| Osteocalcin | 2.25 | 0 | 0 | 1 | 0 | 0 | 2 | 5.25 | Low |
| BALP | 2.25 | 0 | 0.3 | 1 | 0.5 | 0 | 2 | 6.05 | Moderate |
| Dpd | 2.25 | 1 | 0 | 1 | 0 | 0 | 2 | 6.25 | Moderate |
| CTx | 2 | 0 | 0 | 1 | 0 | 0 | 2 | 5 | Low |
| Parathyroid hormone | 2.25 | 1 | 0.3 | 1 | 0.5 | 0 | 2 | 7.05 | Moderate |
| 25(OH)D | 2.25 | 1 | 0.3 | 1 | 0.5 | 0 | 2 | 7.05 | Moderate |
| IGF-1 | 2.25 | 1 | 0 | 1 | 0 | 0 | 2 | 6.25 | Moderate |
| Height | 2.25 | 1 | 0.8 | 1 | 1 | 0 | 2 | 8.05 | High |
